# Supplementary material for: OpenMM 7: Rapid development of high performance algorithms for molecular dynamics
Source: PLoS Comput Biol. 2017 Jul 26;13(7):e1005659. doi: 10.1371/journal.pcbi.1005659 (PMC5549999; doi:10.1371/journal.pcbi.1005659)
Supplement: S1 Source Code — (ZIP) [file pcbi.1005659.s002.zip › OpenMM-7.0.1-Source/docs/api-python/generated/simtk.openmm.openmm.CMAPTorsionForce.html]

CMAPTorsionForce — OpenMM 7.0.1.dev-5e86c4f documentation


# CMAPTorsionForce¶

*class* `simtk.openmm.openmm.``CMAPTorsionForce`(*\*args*)¶
:   This class implements an interaction between pairs of dihedral angles. The interaction energy is defined by an “energy correction map” (CMAP), which is simply a set of tabulated energy values on a regular grid of (phi, psi) angles. Natural cubic spline interpolation is used to compute forces and energies at arbitrary values of the two angles.

    To use this class, first create one or more energy correction maps by calling addMap(). For each one, you provide an array of energies at uniformly spaced values of the two angles. Next, add interactions by calling addTorsion(). For each one, you specify the sequence of particles used to calculate each of the two dihedral angles, and the index of the map used to calculate their interaction energy.

    `__init__`(*self*) → CMAPTorsionForce¶
    :   \_\_init\_\_(self, other) -> CMAPTorsionForce

        Create a CMAPTorsionForce.

    Methods

    |  |  |
    | --- | --- |
    | `__init__`((self) -> CMAPTorsionForce) | \_\_init\_\_(self, other) -> CMAPTorsionForce |
    | `addMap`((self, size, energy) -> int) | Create a new map that can be used for torsion pairs. |
    | `addTorsion`((self, map, a1, a2, a3, a4, b1, ...) | Add a CMAP torsion term to the force field. |
    | `getForceGroup`((self) -> int) | Get the force group this Force belongs to. |
    | `getMapParameters`(self, index) | Get the energy values of a map. |
    | `getNumMaps`((self) -> int) | Get the number of maps that have been defined. |
    | `getNumTorsions`((self) -> int) | Get the number of CMAP torsion terms in the potential function |
    | `getTorsionParameters`(self, index) | Get the force field parameters for a CMAP torsion term. |
    | `setForceGroup`(self, group) | Set the force group this Force belongs to. |
    | `setMapParameters`(self, index, size, energy) | Set the energy values of a map. |
    | `setTorsionParameters`(self, index, map, a1, ...) | Set the force field parameters for a CMAP torsion term. |
    | `updateParametersInContext`(self, context) | Update the map and torsion parameters in a Context to match those stored in this Force object. |
    | `usesPeriodicBoundaryConditions`((self) -> bool) | Returns whether or not this force makes use of periodic boundary conditions. |

    `getNumMaps`(*self*) → int¶
    :   Get the number of maps that have been defined.

    `getNumTorsions`(*self*) → int¶
    :   Get the number of CMAP torsion terms in the potential function

    `addMap`(*self*, *size*, *energy*) → int¶
    :   Create a new map that can be used for torsion pairs.

        |  |  |
        | --- | --- |
        | Parameters: | - **size** (*int*) – the size of the map along each dimension - **energy** (*vector< double >*) – the energy values for the map. This must be of length size\*size. The element energy[i+size\*j] contains the energy when the first torsion angle equals i\*2\*PI/size and the second torsion angle equals j\*2\*PI/size. |
        | Returns: | the index of the map that was added |
        | Return type: | int |

    `getMapParameters`(*self*, *index*)¶
    :   Get the energy values of a map.

        |  |  |
        | --- | --- |
        | Parameters: | **index** (*int*) – the index of the map for which to get energy values |
        | Returns: | - **size** (*int*) –   the size of the map along each dimension - **energy** (*vector< double >*) –   the energy values for the map. This must be of length size\*size. The element energy[i+size\*j] contains the energy when the first torsion angle equals i\*2\*PI/size and the second torsion angle equals j\*2\*PI/size. |

    `setMapParameters`(*self*, *index*, *size*, *energy*)¶
    :   Set the energy values of a map.

        |  |  |
        | --- | --- |
        | Parameters: | - **index** (*int*) – the index of the map for which to set energy values - **size** (*int*) – the size of the map along each dimension - **energy** (*vector< double >*) – the energy values for the map. This must be of length size\*size. The element energy[i+size\*j] contains the energy when the first torsion angle equals i\*2\*PI/size and the second torsion angle equals j\*2\*PI/size. |

    `addTorsion`(*self*, *map*, *a1*, *a2*, *a3*, *a4*, *b1*, *b2*, *b3*, *b4*) → int¶
    :   Add a CMAP torsion term to the force field.

        |  |  |
        | --- | --- |
        | Parameters: | - **map** (*int*) – the index of the map to use for this term - **a1** (*int*) – the index of the first particle forming the first torsion - **a2** (*int*) – the index of the second particle forming the first torsion - **a3** (*int*) – the index of the third particle forming the first torsion - **a4** (*int*) – the index of the fourth particle forming the first torsion - **b1** (*int*) – the index of the first particle forming the second torsion - **b2** (*int*) – the index of the second particle forming the second torsion - **b3** (*int*) – the index of the third particle forming the second torsion - **b4** (*int*) – the index of the fourth particle forming the second torsion |
        | Returns: | the index of the torsion that was added |
        | Return type: | int |

    `getTorsionParameters`(*self*, *index*)¶
    :   Get the force field parameters for a CMAP torsion term.

        |  |  |
        | --- | --- |
        | Parameters: | **index** (*int*) – the index of the torsion for which to get parameters |
        | Returns: | - **map** (*int*) –   the index of the map to use for this term - **a1** (*int*) –   the index of the first particle forming the first torsion - **a2** (*int*) –   the index of the second particle forming the first torsion - **a3** (*int*) –   the index of the third particle forming the first torsion - **a4** (*int*) –   the index of the fourth particle forming the first torsion - **b1** (*int*) –   the index of the first particle forming the second torsion - **b2** (*int*) –   the index of the second particle forming the second torsion - **b3** (*int*) –   the index of the third particle forming the second torsion - **b4** (*int*) –   the index of the fourth particle forming the second torsion |

    `setTorsionParameters`(*self*, *index*, *map*, *a1*, *a2*, *a3*, *a4*, *b1*, *b2*, *b3*, *b4*)¶
    :   Set the force field parameters for a CMAP torsion term.

        |  |  |
        | --- | --- |
        | Parameters: | - **index** (*int*) – the index of the torsion for which to set parameters - **map** (*int*) – the index of the map to use for this term - **a1** (*int*) – the index of the first particle forming the first torsion - **a2** (*int*) – the index of the second particle forming the first torsion - **a3** (*int*) – the index of the third particle forming the first torsion - **a4** (*int*) – the index of the fourth particle forming the first torsion - **b1** (*int*) – the index of the first particle forming the second torsion - **b2** (*int*) – the index of the second particle forming the second torsion - **b3** (*int*) – the index of the third particle forming the second torsion - **b4** (*int*) – the index of the fourth particle forming the second torsion |

    `updateParametersInContext`(*self*, *context*)¶
    :   Update the map and torsion parameters in a Context to match those stored in this Force object. This method provides an efficient method to update certain parameters in an existing Context without needing to reinitialize it. Simply call setMapParameters() and setTorsionParameters() to modify this object’s parameters, then call updateParametersInContext() to copy them over to the Context.

        The only information that can be updated with this method is the energy values for a map, and the map index for a torsion. The size of a map and the set of particles involved in a torsion cannot be changed. Also, new bonds and torsions cannot be added.

    `usesPeriodicBoundaryConditions`(*self*) → bool¶
    :   Returns whether or not this force makes use of periodic boundary conditions.

        |  |  |
        | --- | --- |
        | Returns: | false |
        | Return type: | bool |

    `__delattr__`¶
    :   x.\_\_delattr\_\_(‘name’) <==> del x.name

    `__format__`()¶
    :   default object formatter

    `__getattribute__`¶
    :   x.\_\_getattribute\_\_(‘name’) <==> x.name

    `__hash__`¶

    `__reduce__`()¶
    :   helper for pickle

    `__reduce_ex__`()¶
    :   helper for pickle

    `__sizeof__`() → int¶
    :   size of object in memory, in bytes

    `__str__`¶

    `getForceGroup`(*self*) → int¶
    :   Get the force group this Force belongs to.

    `setForceGroup`(*self*, *group*)¶
    :   Set the force group this Force belongs to.

        |  |  |
        | --- | --- |
        | Parameters: | **group** (*int*) – the group index. Legal values are between 0 and 31 (inclusive). |

High performance molecular simulation on GPUs


### Navigation

- Application Layer
- Library Layer
  - Core Objects
  - Forces
  - Integrators
  - Extras

©2015, Stanford University and the Authors.
|
Powered by Sphinx 1.3.1
& Alabaster 0.7.7
|
Page source
